# Impact of internal and external factors on prosocial choices in rhesus macaques

**DOI:** 10.1098/rstb.2019.0678

**Published:** 2021-01-11

**Authors:** Jérôme Sallet, Andrew Emberton, Jessica Wood, Matthew Rushworth

**Affiliations:** 1 Wellcome Integrative Neuroimaging Centre, Department of Experimental Psychology, Oxford, OX1 3SR, UK; 2 Biomedical Sciences Services, University of Oxford, Oxford, OX1 3SR, UK; 3 Univ Lyon, Université Lyon 1, Inserm, Stem Cell and Brain Research Institute U1208, Bron, France

**Keywords:** prosociality, reward, social status, cost, rhesus macaque

## Abstract

While traditional economic models assume that agents are self-interested, humans and most non-human primates are social species. Therefore, many of decisions they make require the integration of information about other social agents. This study asks to what extent information about social status and the social context in which decisions are taken impact on reward-guided decisions in rhesus macaques. We tested 12 monkeys of varying dominance status in several experimental versions of a two-choice task in which reward could be delivered to self only, only another monkey, both the self and another monkey, or neither. Results showed dominant animals were more prone to make prosocial choices than subordinates, but only when the decision was between a reward for self only and a reward for both self and other. If the choice was between a reward for self only and a reward for other only, no animal expressed altruistic behaviour. Finally, prosocial choices were true social decisions as they were strikingly reduced when the social partner was replaced by a non-social object. These results showed that as in humans, rhesus macaques' social decisions are adaptive and modulated by social status and the cost associated with being prosocial.

This article is part of the theme issue ‘Existence and prevalence of economic behaviours among non-human primates’.

## 1. Introduction

Most economic models rely on the assumption that agents are self-interested [1]. However, many of our decisions are made in a social environment. Evaluating the options at stake is often done while integrating this social dimension—for example, when deciding to take or to share the last piece of pie at a family dinner. But the social dimension can also sometimes be ignored. This was the case when customers could not resist stock piling toilet paper at the time of the COVID-19 pandemic, irrespective of the potential need of others.

The importance of the social environment is such that computational needs associated with living in large and complex social groups are thought to be a key factor that has driven the expansion of the neocortex in primates [2]. Social information in humans is thought to be supported by a set of brain regions often called the social brain [3,4]. Neuroanatomical and functional imaging studies have shown that the building blocks of the human social brain have been present since the last common ancestor of humans and old-world monkeys [5–7] approximately 25 Ma [8]. Some evidence suggests that it could even have been present since the last ancestor of humans and new-world monkeys [9,10], around 35 Ma [8]. Altogether these studies are establishing that non-human primates are a good model for investigating the neuronal basis for social cognition.

Like most primate species, rhesus macaques are social creatures. Social information impacts on their decisions. For example, it can distract them from reaching to pick up food items [11,12]. They may even sacrifice juice reward to watch social stimuli [13]. In addition to showing a preference for social information, it is clear that rhesus macaques use such information to make inferences about others' knowledge of the world [14–16], to guide their decision about where to pay attention [17], or which options to select in cooperative/competitive reward-based decision tasks [18–20]. Social presence has also been shown to facilitate learning [21]. But the social environment is not always perceived as positive. For instance, neurons in the medial orbitofrontal cortex that are sensitive to reward value are modulated negatively when a juice reward is delivered in a social context compared to a non-social context [22].

Many factors have been shown to impact on prosocial decisions, i.e. decisions that would benefit others. Two categories of factors could be distinguished: factors related to the social agents itself (e.g. its social status), or factors related to the environment the social agent is interacting with (e.g. the rewards at stake, the behaviour of a social partner). We refer to these two factors as internal and external factors, respectively. In humans, internal factors such as social status are thought to modulate social behaviours, as assessed by surveys of the population [23,24] or from artificial settings in which social status is manipulated [25]. However, social status is a complex parameter in human society and some studies are questioning results showing that low social status is associated with a relatively higher tendency to show prosocial behaviour than high social status [26]. In non-human primates, social status is associated with how dominant an individual is within a social group. While social status in humans could be inferred from surveys [23,24], in non-human primates researchers rely notably on the direction of agonistic interactions to establish how an individual ranks within a social group [27–29]. Dominance is a key factor that governs the organization of the despotic rhesus macaque society [30]. Among primates a high social status is often associated with prior access to resources, reduced stress level, and better health status [29,31–34]. During social foraging, high-ranked capuchin monkeys occupied a central spatial position in the group. Although it decreases their probability of discovering new food sources, it increases their chances of parasitizing food sources discovered by other individuals [35]. In this context, dominant capuchins are using others’ foraging skills to their own benefits. In an opposite manner to what has been observed in humans or in capuchins, dominant long-tailed macaques have been shown to be relatively more prosocial than subordinate individuals [36].

Factors external to the individuals also impact on social decisions. The scarcity of preferred food items reduces social tolerance within japanese macaque social groups. As a consequence, small food patches result in higher social dispersion and differential foraging strategies for dominants and subordinates [37]. In laboratory experiments, manipulating reward contingencies has been shown to be alter prosocial tendencies. Macaque rhesus monkeys chose to reward others if the alternative option was a lack of reward for both, but preferred to choose a reward for self when it was the alternative option [38]. Experiments in apes have also shown that chimpanzees too could make choices that reward others. However, their prosocial decisions principally reflect a mutualistic maximization of rewards. They were prosocial if they too were rewarded [39,40]. Finally, rhesus macaques have been shown to be sensitive to whether rewards are shared equally or not between partners [41]. When prosocial choices are costly (i.e. no reward for self), animals prefer to be selfish rather than altruistic (i.e. reward for other only). The value of the partner or its behaviour could be important too. For example, rhesus macaques value more highly the faces of dominant than of subordinate monkeys, and therefore would sacrifice more juice reward to watch the former [13]. Partners' behaviour could also modify how prosocial an animal is. In apes, prosocial decisions are reduced by noisy, begging partners [42]. Rhesus macaques are more likely to behave cooperatively in a Prisoner's Dilemma game if the partner has made a cooperative choice on the previous trial [19]. Reciprocity has also been observed in contexts in which long-tailed macaques withhold delivering a punishment [43].

Altogether, reward-based decisions in a social context can be impacted by many parameters. Beyond a sensitivity to the social information, the diversity of the social effects on prosocial decisions could reflect either differences in the context in which decisions are made, or the species studied. In this study we examined in a two-choice task how several internal and external parameters modulated prosocial decisions. We tested to what extent dominance status determines how prosocial rhesus macaques are. We also tested how several external factors could impact on prosocial decisions. More specifically, we manipulated the nature of the partner (social versus non-social), the identity of the social partner and its behaviour. We also manipulated the cost of being social by rewarding the partner first or by only rewarding the partner. Altogether, these manipulations showed that macaque rhesus social decisions are adaptive.

## 2. Material and methods

### (a) Subjects

Twelve rhesus macaques (*Macaca mulatta*) (six males, six females; 7.21 years old ± 1.20 s.d.; table 1) participated in the first study (Study 1). Eight of those subjects were enrolled in the second study (Study 2). All animals but two lived in social group, of two to five animals. Social groups were determined based on assignments to research groups and research projects. O1 and O2 were temporarily single-housed for husbandry reasons around the time of Study 1. O1, O2 and P5 had been reassigned to new social groups by the time of Study 2.

**Table 1.** Demographic information relative to subjects involved in Study 1 and Study 2. No social status information was available for O1 and O2 at the time of Study 1. The study occurred during a volatile time for the group that O1 and O2 lived in and they were temporarily single-housed for husbandry reasons. P7, O4, P2, and P1 did not take part in Study 2.

ID	sex	age (years)	social status (Study 1)	social status (Study 2)
P6	F	8.87	−84.38	−84.38
S1	F	7.17	−70.37	−70.37
P7	F	8.03	−31.82	—
P4	M	5.96	−31.03	−31.03
O4	F	7.36	−13.89	—
O2	M	6.59	—	27.27
O1	M	6.52	—	21.43
P2	F	6.02	31.25	—
P3	M	6.04	54.55	54.55
P5	M	8.71	58.33	31.82
P1	F	6.08	59.09	—
O3	M	9.12	63.64	63.64

### (b) Social status

Social status, or dominance, was assessed through behavioural observations of agonistic interactions in their home cages/pen, using an ethogram developed for previous studies [27,44]. It consisted of 10-min observation sessions. The first 5 min were considered as an habituation phase; behavioural scoring was only conducted in the second 5 min of the session. More than 10 behavioural sessions were collected per animal. Data collection started prior to the beginning of Study 1 and continued throughout the experiment.

**Figure 1.**
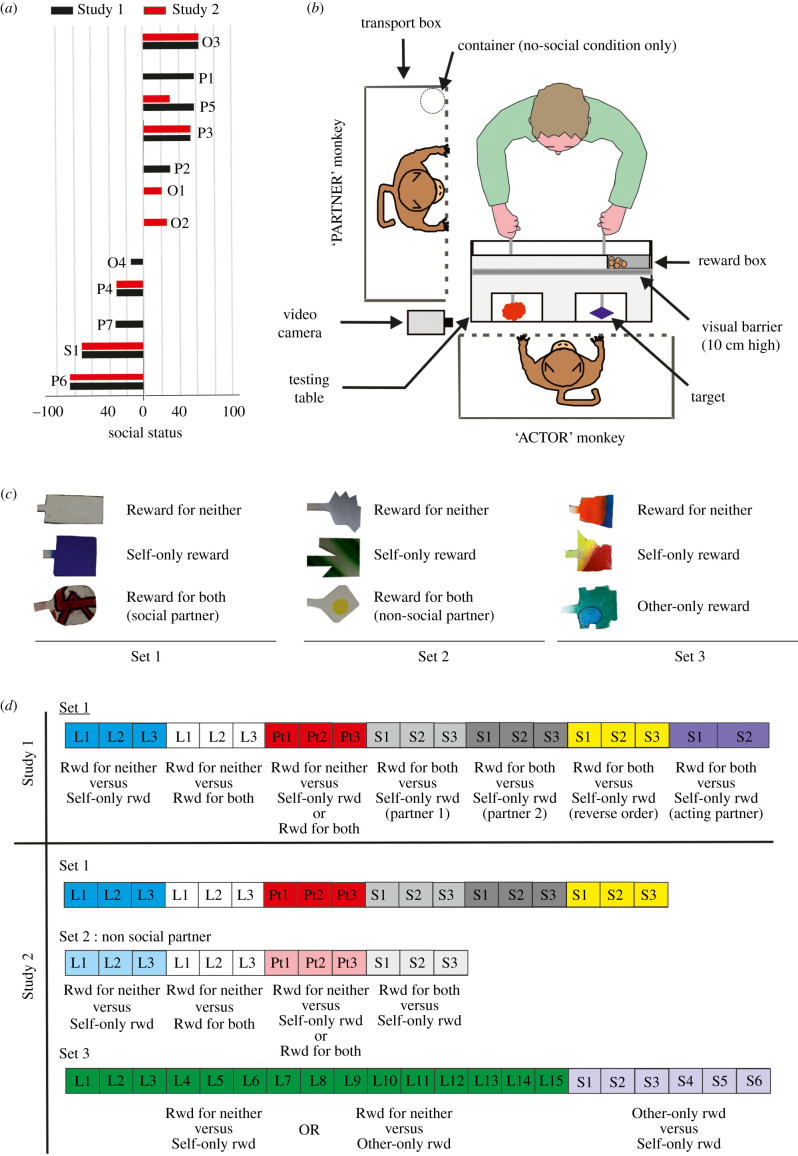
(*a*) Social status of the animals tested in Study 1 (black bars) and Study 2 (red bars). (*b*) Experimental set-up used for Study 1 and Study 2. (*c*) Sets of targets used in the different experimental conditions employed with the animals. Targets within a set differed along two dimensions (shape and colour). (*d*) Theoretical timeline of the Studies 1 and 2. Each square represents a testing day. The different colour represents the different phases/experimental condition. L, learning session; Pt, pre-testing session; S, session; T, training session; Rwd, reward.

Dominance was calculated as the difference between dominant behaviours minus submissive behaviours divided by the number of social interactions (dominant, submissive and neutral) displayed by the individual. Dominance scores for each animal are presented in figure 1*a*. Importantly, we did not observe a significant correlation between age and social status (*r* = −0.14, *p* = 0.71).

### (c) Partner

All partners were adult rhesus monkeys. Partners were animals living in the same room and were, therefore, familiar social agents. The recruitment was constrained by their assignments to other experiments conducted in parallel in the laboratory at the time [45–48]. Those two factors only guided our selection of partners.

### (d) Set-up

During training and testing sessions, the subject sat inside a transport box (62 × 52 × 45 cm) next to a homemade testing table (figure 1*b*). The experimenter stood on the opposite side of the testing table and, on each trial, presented the monkey simultaneously with two wooden targets. Targets were hidden from the monkey's sight during the inter-trial interval (ITI), in the mid-section of the hollow testing set-up. Three sets of targets and different reward schedules were used during the experiments (figure 1*c*). A visual barrier on the table prevented the monkey from seeing rewards he could potentially receive.

A second transport box of similar dimension was always placed to the left of the actor monkey. In the social condition, a monkey sat inside the second box for the duration of the experiment. Both animals could see each other but could not interact with each other. In the non-social condition, the social partner was replaced by an empty opaque container that was placed inside the partner's transport box.

### (e) Experimental protocol

In each session of the different versions of this 2-choice task, the ‘actor’ monkey was asked to perform 40 trials. Two targets were presented simultaneously, and the subject had 20–25 s to make a choice by moving its hand into the slot of the chosen target. The completion of a choice was confirmed by an auditory feedback. Both targets were then withdrawn. In the case of a rewarded trial, a food reward was placed in the slot corresponding to the selected target (note that for P7, and for the experimental condition associated with Set 3, the unselected target was withdrawn but the selected target was left in position until the reward was delivered). The food reward given was either a chocolate peanut or a chocolate raisin, depending on the preference of the ‘actor’ monkey. If a prosocial choice was made, a reward was then given to the social or non-social partner in the second box. Each trial was followed by a 5–10 s inter-trial interval.

If the animal touched both targets, the trial was aborted and no feedback was delivered; the aborted trial was repeated at the end of the session. If the ‘actor’ did not produce a response within 20–25 s, then the targets were withdrawn and the trial was repeated at the end of the session. If this happened for more than five consecutive trials, the session was stopped. Aborted sessions were discarded from the analysis.

Several experimental conditions were tested in two studies. At least two months separated the two studies. Experimental manipulations were conducted in the same order as presented below with all the monkeys (figure 1*d*). During the training phase, the animal was first trained to discriminate between a target associated with a reward for self (referred as self-only-reward target) and target associated with reward to neither individual (referred to as the reward-for-neither target). Once they reached 80% correct performance for three sessions, the prosocial rewarding target (a target associated with reward both for the actor and the other monkey in the second box—referred to as the reward-for-both target) was introduced, and its value was learned in trials in which it was pitted against the reward-for-neither target. The same criterion was used. Once the animals had learned the value of these second rewarded options, they had to perform over 80% correct over three sessions, with both pairs being randomly interleaved during each session. These pre-testing sessions were done to minimize a potential recency effect. Then, the animal was asked to choose between the self-only target and the Reward-for-Both target for three consecutive sessions with a given social partner (Partner 1), and then for three consecutive sessions with a different social partner (Partner 2). We referred to these sessions as the standard condition. In the following three sessions, the order in which rewards were delivered was altered. In this so-called ‘reverse order’ condition, a prosocial choice resulted in the reward being given to the social partner before the actor; a selfish decision resulted in the reward being given to the actor after an approximately 3–4 s delay. In the last condition of Study 1 (referred to as the selfish partner condition), the partner was asked to make five selfish decisions every five trials. On the blocks, the partner was asked to make decisions, the testing set-up was turned 90° and positioned in front of the partner's transport box. The self-only-reward target was the only target presented to the partner, either to the left or the right slot. Once touched by the partner, an auditory feedback was provided. The target was then withdrawn, and a food reward was placed in the corresponding slot. The actor could clearly hear the reward-related auditory feedback and then see the reward being given only to the partner. Each trial was followed by a 5–10 s inter-trial interval. For that specific manipulation, the 120 trials were collected over two sessions. For the purpose of the analysis, the trials were split into blocks of 40 trials to match up other conditions.

With the exception of the last condition, Study 2 was, first of all, a replication of Study 1. We then trained the animals with the same training protocol used for Set 1 to discriminate targets from Set 2. With this second set of targets, we aimed to test the social nature of the prosocial decisions by replacing the social partner with a non-social partner (an empty opaque container in the second box). Training and testing schedules conducted with Set 2 targets were similar to those used for the standard conditions with Set 1 and described above.

A third set of targets (Set 3) was used for the final experimental condition of Study 2 (referred to as the altruistic condition). Each session was 40 trials long. Animals were trained to discriminate between a new self-only-reward target versus a reward-for-neither target. They were also presented with a new type of option, a target associated with reward for the partner only (referred as an other-only-reward option). Animals learned about this option in alternating blocks of five trials in which the other-only-reward option was pitted against the reward-for-neither. In the other block of five trials, the animal was offered choices between the self-only-reward target and the reward-for-neither target. This procedure was employed to maintain the motivation of the animals to perform choices in blocks in which they would not receive any reward. This learning phase lasted for 15 sessions. No performance criterion was used for this experimental condition but all animals performed above 75% correct for the self-only-reward versus reward-for-neither trials. Animals were then given choices between a self-only-reward option and an other-only-reward option for six sessions.

### (f) Analysis

Analysis was focused on the performance of the animals. Statistical analyses conducted in SPSS were repeated-measures analyses of variance (ANOVAs), with Greenhouse–Geisser correction applied when sphericity could not be assumed (Mauchly's *W*, *p* < 0.05). *Post hoc* tests were Bonferroni corrected for multiple comparison. The dominance status of the animal was included as a covariate in our analysis. Comparison with statistical chance levels was based on the work by Steffens and colleagues [49]. Repeated-measures correlation was conducted using rmcorr toolbox in R [50].

## 3. Results

### (a) Study 1

**Figure 2.**
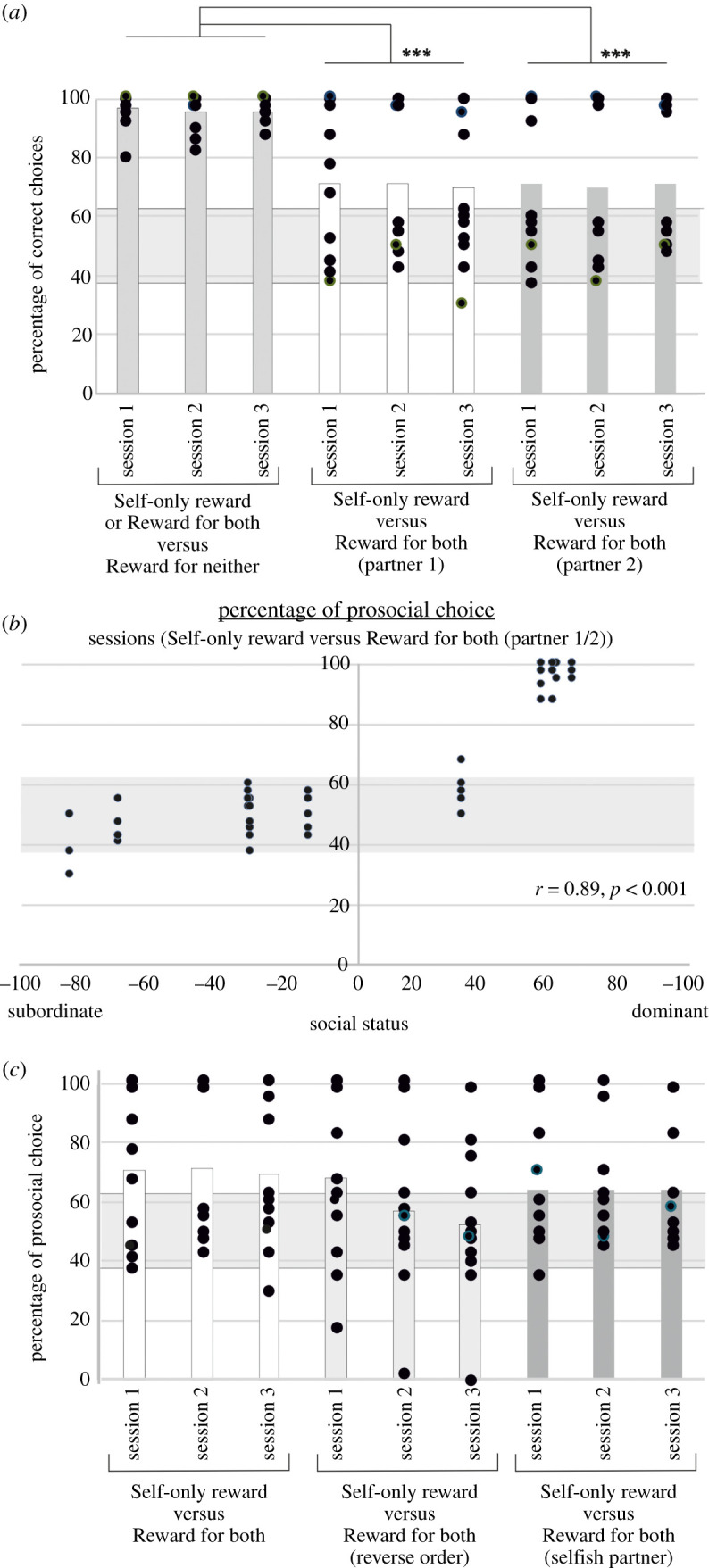
Behavioural performance in the prosocial task (Study 1). (*a*) Performance measured in three experimental conditions. Each condition was repeated for three consecutive sessions. First, the animals were asked to make a choice between a ‘Self-only-reward’ option and a ‘Reward-for-neither’ option, or a choice between a ‘Reward-for-both’ option and a ‘Reward-for-neither’ option. They were then asked to choose between a ‘Self-only-reward’ option and a ‘Reward-for-both’ option, with a first and a second partner (data shown separately for each individual animal's encounters with each of the two partners). For the purpose of the analysis, we considered the prosocial choice (‘Reward-for-both’ option) as the correct choice. ***p < 0.001 from the Bonferroni *post hoc* comparison between experimental conditions. (*b*) Impact of social status on prosocial decisions. Prosocial choices were obtained from the six sessions in which animals were asked to choose between a ‘Self-only-reward’ option versus a ‘Reward-for-both’ option. (*c*) Following the task manipulations presented in panel (*a*), the animals were tested in two additional experimental environments. First, the order in which the reward was delivered was inverted, so the Partner in the second box would now receive their reward before the actor. In a final task manipulation, we interleaved blocks of five trials in which the actor made choices, and five trials in which the partner made choices. In the latter case, the partner was presented with a single option, the ‘Self-only-reward’ option. Therefore, the partner was constrained to always choose selfishly and so this condition is referred to as the ‘selfish partner’ condition. Performances are compared with the first three sessions of the ‘Self-only-reward’ option versus ‘Reward-for-both’ trials. Black dot represent performance recorded per session and per animal. The grey areas on panels 2*a*, *b* and *c* represent statistical chance levels [49].

Animals were first trained to learn the value associated with each target until they reached a criterion of 80% of correct choices for three sessions discriminating between self-only reward versus reward-for-neither and for three sessions discriminating reward-for-both versus reward-for-neither. Then the targets were combined for three pre-testing sessions in which the animals had to choose between a rewarded option and a non-rewarded one. In these sessions, the rewarded option was either a self-only-reward option or a reward-for-both option. All 12 animals performed above 90% correct choices when asked to discriminate between a rewarded option and a non-rewarded (95.86% ± 5.32 s.d.), even if the rewarded option also meant rewarding a ‘partner’ monkey (figure 2*a*).

Finally, ‘actor’ monkeys were asked to choose between a self-only-reward option and a reward-for-both option. Three sessions were completed with one partner (partner 1), and three sessions were then conducted with a second partner (partner 2) to check whether the identity of the partner could impact of the actor's prosociality.

First, we compared performance using a repeated-measure ANOVA including experimental conditions (pre-testing; testing with partner 1; testing with partner 2) and sessions (three sessions per experimental condition) as within-subject factors. Social status was included as a covariate factor. For our analysis, we assumed that the prosocial choice was the correct choice.

We observed a significant effect of the experimental condition (*F*_2,16_ = 5.585, *p* = 0.012). This effect was driven by the fact that the animals made significantly fewer correct choices when the alternative option was equally rewarding (testing sessions with partner 1 or 2) as compared to when the alternative option led to a reward for neither (pre-testing sessions) (Bonferroni corrected *post hoc* analysis, *p* < 0.05). On average, monkeys chose the prosocial option in 70.72% ± 24.35 s.d. of the trials in the testing sessions (figure 2*a*). Importantly, we observed a considerable variability in performance. Compared with statistical chance levels [49], we identified two types of monkeys: animals that were prosocial and animals that randomly chose one or the other option (figure 2*a*). We did not observe an effect of sessions (*F*_2,16_ = 0.49, *p* = 0.952) or an interaction between experimental condition and sessions (*F*_2,16_ = 0.850, *p* = 0.504). The identity of the partner did not influence the variability in the results either (Bonferroni corrected *post hoc* analysis, *p* > 0.05). A repeated-measure correlation analysis between performances recorded in sessions with partner 1 and sessions with partner 2 confirmed that the partner identity did not impact on animals' choices (*r* = 0.8774, *p* < 0.001). The performance in these six sessions was instead correlated with the social status of the actor animals (*r* = 0.89, *p* < 0.001) (figure 2*b*).

Second, we manipulated the social environment to assess how external factors could impact on how prosocial animals acted. For these manipulations we kept using the same targets and no re-training was conducted. The first manipulation consisted of changing the order in which rewards was delivered to the animals. Initially, the reward to the actor was delivered first, followed by the reward delivery to the partner. However, in the reversed order sessions, the order partner was rewarded before the actor. To control for a potential delay discounting effect, we added a short delay of 3–4 s between choice and outcome delivery for the selection of the self-only-reward target. Second, in selfish partner sessions, we restored the original reward delivery order and timings, but we asked the partner monkey to make responses that were constrained to be of selfish nature for the partner. Every five trials, the partner was asked to touch the self-only-reward target for five trials. This meant that the partner had to behave selfishly. We were expecting that those manipulations would make the ‘actor’ less prosocial. Three sessions were conducted for each manipulation. Performance was compared with the first three sessions the Actor performed in the standard version of the task (three standard sessions with partner 1). A repeated-measure ANOVA was conducted with experimental condition (three levels: standard version; reverse order; selfish partner) and sessions (three sessions per experimental condition) as within-subject factors. Social status was included as a covariate factor. The different experimental conditions had no significant effect on performance (Greenhouse–Geisser, *F*_2,16_ = 1.437, *p* = 0.511). On average, animals were prosocial in 70.7% ± 24.4 s.d. of the trials, versus 59.4% ± 26.6 s.d. and 64.2% ± 20.0 s.d. in our two altered versions of the task. We did not observe an effect of sessions (Greenhouse–Geisser, *F*_2,16_ = 1.020, *p* = 0.23) or an interaction between experimental condition and sessions (Greenhouse–Geisser, *F*_2,16_ = 1.864, *p* = 0.172).

### (b) Study 2

**Figure 3.**
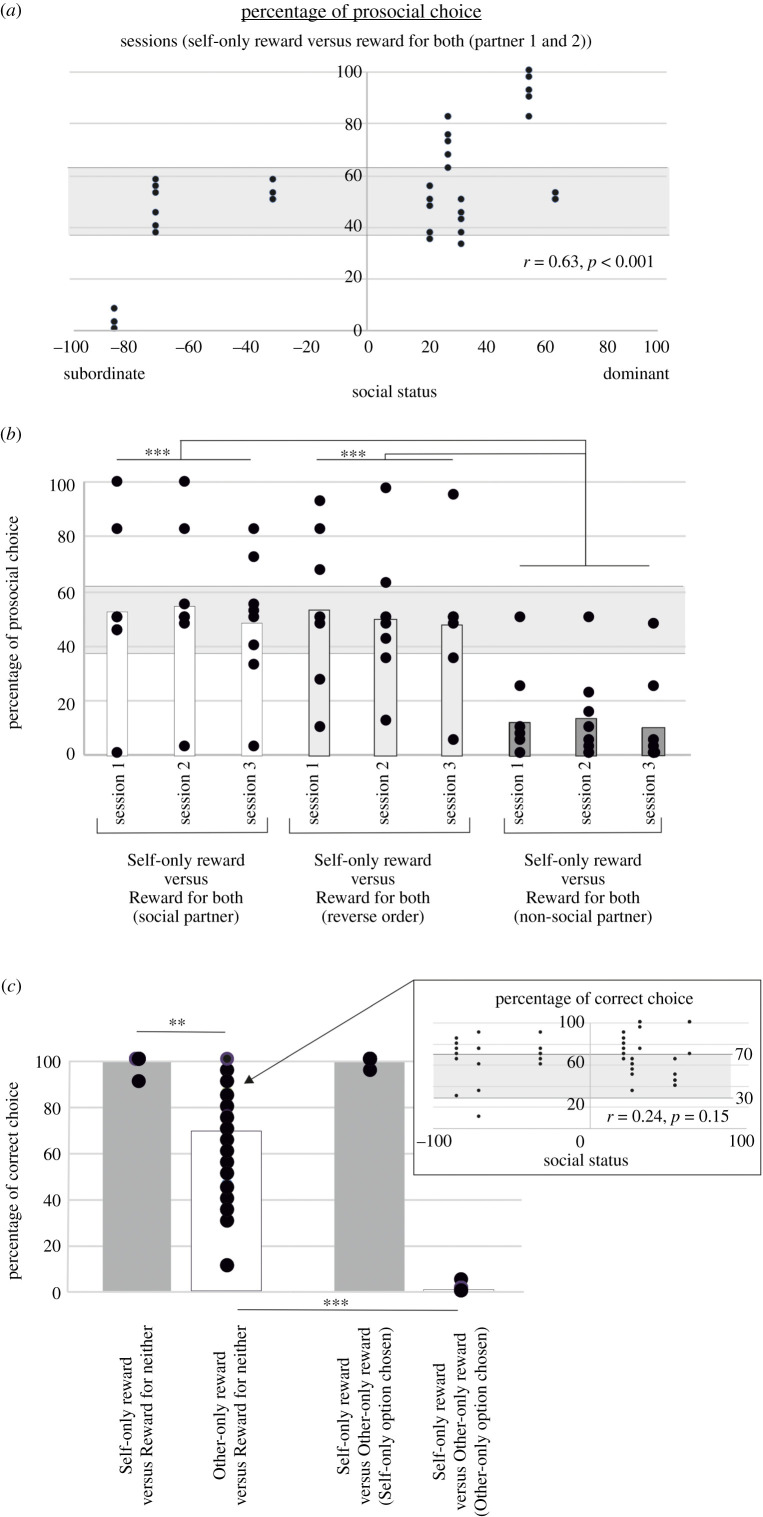
Behavioural performance in the prosocial tasks (Study 2). (*a*) Impact of social status on prosocial decisions. Data points were obtained from the six sessions in which animals were asked to choose between a self-only-reward option versus a reward-for-both option. (*b*) Impact of task manipulations on prosocial choices. The animals were presented with two modified experimental environments. First, the order in which the reward was delivered was inverted, so the partner would now receive their reward before the actor. In a second task manipulation, we replaced the social partner with an empty container. The container was placed in the second box where the partner had previously been sat. The social rewards that were delivered were placed in this opaque container. Performances are compared with those in the first three sessions of trials self-only-reward option versus reward-for-both option decisions that each subject performed. ***p < 0.001 from the Bonferroni *post hoc* comparison between experimental conditions. (*c*) Altruistic choice. In a final experimental condition, animals were asked to discriminate in interleaved blocks of five trials in which either the self-only-reward option was pitted against a reward-for-neither option or in which an other-only-reward option was pitted against a reward-for-neither option. The animals were trained for 15 sessions before being asked, for six sessions, to choose between a self-only-reward option and an other-only-reward option. Performances were compared with those in the last six sessions of the training period. ***p < 0.001, **p < 0.01 from the repeated-measure ANOVA analyses. Black dots represent performance recorded per session and per animal. The grey areas on panels 3*a*–*c* represent statistical chance levels [49].

Eight animals from study 1 participated in this second study. First of all, we could replicate the effects that we had previously reported using a similar training and testing protocol. As some animals had been reassigned to different social groups, their social status had changed (figure 1*a*). When assuming that the prosocial choice was the correct choice, the animals made almost no mistake in the three pre-testing sessions (97.7% ± 4.01 s.d.). Their performance dropped when the alternative option was equally rewarding compared to when the alternative option led to a reward for neither, as it was in the pre-testing sessions (Greenhouse–Geisser, *F*_2,12_ = 31.098, *p* = 0.001; data not shown). In the standard sessions, animals made fewer prosocial choices (50.9% ± 25.4 s.d.) than they did in Study 1 (Greenhouse–Geisser, *F*_1,7_ = 6.98, *p* = 0.033). Nevertheless, an overall similar pattern of results was observed when performance was compared with statistical chance levels [49]; some animals were prosocial (above 62.5% correct choices) and others performed at chance level (figure 3*a*). P6, who was choosing the prosocial option at chance level in Study 1, was reluctant to choose this option in Study 2, being the sole animal to be selfish. We did not observe a main effect of sessions (Greenhouse–Geisser, *F*_2,12_ = 0.779, *p* = 0.446) or an interaction between experimental condition and sessions (Greenhouse–Geisser, *F*_2,12_ = 1.716, *p* = 0.224). Finally, we confirmed that the identity of the partner was not a factor influencing animals' choices (Bonferroni corrected *post hoc* analysis, *p* > 0.05). Once again, repeated-measures correlation analysis confirmed that variability in prosocial tendency in standard sessions was indeed not modulated by partner's identity (*r* = 0.80, *p* < 0.001) but was positively correlated with the social status of the actor animals (*r* = 0.63, *p* < 0.001) (figure 3*a*).

We then tested, as we did in Study 1, whether the lack of effect of reversing the order of reward delivery could be replicated. We also trained animals with a new set of targets (Set 2; figure 1*d*). With these new targets, the reward-for-both option meant that the social reward would be delivered to a non-social partner—an inanimate object in the second transport box. During the task, social rewards were placed in this container. Performances from these two additional experimental conditions were compared with the first three sessions the actor performed with Partner 1, choosing between the self-reward-only option and the reward-for-both option. A repeated-measure ANOVA was used with experimental condition (3 levels: standard condition, reversed order condition, inanimate object partner condition) and session (3 sessions per experimental condition) as within-subject factors and social status as a covariate factor. Unlike in Study 1, task manipulation had a strong impact on prosociality (Greenhouse–Geisser, *F*_2,16_ = 67.28, *p* < 0.001). Prosocial choices were true social decisions as they were strikingly reduced when the social partner was replaced by a non-social object (figure 4*b*). This effect was driven by a dramatic diminution in performance when the social partner was replaced by a non-social partner compared with other experimental conditions (Bonferroni corrected *post hoc* analysis, *p* < 0.001). Reversing the order of self and other reward delivery did not impact on prosociality (Bonferroni corrected *post hoc* analysis, *p* > 0.05).

Finally, we designed a new testing schedule to study whether an increased cost of being prosocial would have an impact on prosocial decisions. In this new schedule, animals were required to choose an other-only reward or self-only reward. In other words, we tested whether rhesus macaques could make altruistic decisions. To train the animals to learn the value of an other-only reward target, we altered our training protocol. Animals learnt the value of a third set of targets (figure 1*d*). Choices were presented in blocks of five trials and two targets were presented per block. Animals either chose between a self-only-reward option versus a reward-for-neither option, or between an other-only reward option versus a reward-for-neither option. After 15 training sessions, the animals were presented, for six consecutive sessions, with the following choices: choosing between a self-only-reward option and an other-only reward option.

The performance in the last six training sessions was examined with a repeated-measure ANOVA with block type (2 levels: self-only-reward option versus reward-for-neither option; reward for both option versus reward-for-neither option) and sessions (six sessions) as a within-subject factors. Social status was used as a covariate factor. Subjects' accuracies when discriminating between a self-only-reward option and a reward-for-neither option was 99.8% ± 1.4 s.d. (figure 3*c*). They were also prone to select the other-only reward option when the alternative was a reward-for-neither option (70.7% ± 22.1 s.d.). Their performance were significantly better in the self-only-reward block than in the other-only reward block (Greenhouse–Geisser, *F*_1,6_ = 20.059, *p* = 0.004). Compared with statistical chance levels [49], we again identified two types of monkeys based on their performance in the other-only-reward versus reward-for-neither block: animals that were prosocial and animals that chose randomly (figure 3*c*). In this case, however, variability in how prosocially animals behaved was not significantly related to the social status of the actor (*r* = 0.21, *p* = 0.16) (figure 3*c*).

When the animals were asked to choose between a self-only reward option and an other-only reward option, no animal displayed a prosocial bias anymore. The selection of the other-only option in these six testing sessions dropped significantly when compared to the animals’ performance in the other-only block of the last six training sessions (Greenhouse–Geisser, *F*_1,6_ = 116.026, *p* < 0.001). Out of the 1920 choices made by the eight animals over the six sessions, only two choices were altruistic (figure 3*c*).

## 4. Discussion

Overall our results confirm that rhesus macaques do indeed make prosocial decisions. In the pre-testing sessions, they all made decisions that led to a reward for self and other if the alternative was a reward to neither. In the testing sessions, animals were then asked to choose between an option associated with a reward to both themselves and another monkey compared to an option that just delivered reward to self. At the group level, their prosociality tendency in this context was variable. This variability was explained by the individual dominance status. The prosocial bias was reduced when the social partner was replaced by an inanimate object. Finally, they all stopped choosing a prosocial option when the cost of being prosocial increased considerably. They were devoid of prosociality when they were given a choice between either rewarding themselves or rewarding another monkey.

Our study confirms, in rhesus macaques, a somehow surprising result regarding the impact of social status on prosocial behaviour in long-tailed macaques [36]. While rhesus and long-tailed macaques live in very hierarchical societies, with dominance ruling prior-access to resources, dominant individuals have been found to be more prosocial than subordinate animals. Dominance in monkeys is not simply about aggression but also relies on the formation of pair-bonds and alliances [51]. A relatively higher prosociality bias in dominant macaques may help them maintain their social status. Other internal factors such as satiety or effort have also been shown to impact on prosocial behaviour [52,53] and inequity aversion [54] in primates. Future studies will aim at testing for similar effects in rhesus macaques.

Our results showed that dominant monkeys could be more prosocial than subordinates, but data from human subjects suggest the opposite pattern, i.e. subjects with low social socioecomic status are more prosocial than subjects with high socioeconomic status [24,25,55]. Because of the similar architecture supporting social cognition in humans and macaques [4], we hypothesize that this effect might reflect distinct organization of human societies. Different social organization in primates has been related to differences in socio-cognitive tasks. While rhesus macaques are known to have a very hierarchical social organization, Tonkean macaques have a more tolerant social organization [30,56]. They have been shown to perform better than rhesus macaques in some social and cognitive tasks [57]. In addition, while dominant humans might be relatively less prosocial, this does not mean that they are insensitive to social information. In fact, dominant humans who do not use aggression to assert their dominance have been shown to rely more on social information, in comparison to subordinate individuals, to guide their decisions in a complex decision-making task [58]. Personality traits in macaques correlate with performance in cognitive tasks [59]. Future studies about social cognition in non-human primates should consider this dimension too.

In addition to social status, we have shown that external factors are also important for modulating prosocial behaviours. The context in which social decisions are made impacts on how social information is represented and used [37,59–61]. In the present case, an increased cost of being prosocial is associated with a reduction of the prosocial bias. If being prosocial is associated with a loss of a reward for self, then rhesus macaques stop being prosocial. We found, however, that when the alternative to an other-only reward is a reward for neither, then macaques were biased towards giving a reward to the other individual. A preference for giving a reward to another individual as opposed to no one has been previously observed in rhesus macaques. Moreover it is increased by local infusion of oxytocin into the amygdala [62]. Other neurotransmitters and neurohormones have been shown to impact on social status [63,64], which we have shown is itself correlated with prosociality. Altogether these results reveal the diversity of the determinants that impact on prosocial behaviour.

In our paradigm, however, neither the identity of the partner monkey nor its behaviour modulated the prosocial tendencies of the actor monkeys. Based on previous studies showing evidence of reciprocity in Prisoner's Dilemma games [19,43], we were expecting that forcing the partner to make selfish decisions would result in a decrease in the prosocial bias of the actor. This was not, however, the case. Despite the auditory feedback used prior to rewarding the partner, it might be possible that the actor did not pay attention to the partner. It is possible that our protocol might have been too simplistic to investigate how prosociality in one individual is modulated by the frequency of prosociality in the partner. It is possible that a lack of change in the actor's behaviour may have reflected the fact that a single common visual stimulus was used as the target before and after the task manipulations were instigated. Therefore, a different protocol varying the order in which the different targets are learned and with different targets for each experimental condition should be considered in future studies. Finally, although kinship, social proximity between actor, and partner or ingroup versus outgroup effects have been shown to impact on social behaviours in apes and rhesus monkeys [43,65], we did not observe within-subject variability in performance in regards to the two familiar partners tested. Using familiar and unfamiliar partners might have resulted in different prosocial bias.

Overall our study shows that prosocial decisions in rhesus macaques are strongly modulated by social status and by some contextual factors. Individuals with a high social status were more prone to choose the prosocial option than individuals with a low social status. When the social partner was replaced by a non-social entity, prosocial decisions were almost abolished. When prosocial decisions were costly, rhesus macaques reduced choosing the prosocial option and even chose exclusively the self-only-reward option if the alternative was an other-only reward. Our results, therefore, suggest that rhesus macaques only make prosocial decisions when these choices will mutually benefit self and others. In line with this lack of altruistic behaviour, studies of group feeding in a competitive context have emphasized punishment from dominant rhesus macaques to subordinates who show interest in food items [66]. This complete lack of altruism in a reward-related context might be a key difference between humans and rhesus macaques. It could reflect the differential evolution of cooperative behaviour in humans and macaques [67]. However, a social behaviour central to the organization of primate societies, social grooming, provides evidence of the existence of a degree of altruistic behaviour in monkeys and apes, albeit in a form that involves a very specific and somewhat stereotyped behaviour [68]. Social grooming could occupy up to 20% of a non-human primate's daily activity and has been related to the release of endorphins [69]. Therefore, an alternative explanation for the lack of altruism in a foraging context could be linked to the type of social organization typically observed in rhesus macaques. Rhesus macaques are known for their tyrannical societies; other macaque species exhibit different patterns of social organization [30,56]. Behavioural experiments with subjects from more egalitarian macaque societies such as those of the Sulawesi or Tonkean macaques would help to resolve this issue. For instance, Tonkean macaques have better performance at pointing out a baited location to an experimenter than rhesus, barbary or long-tailed macaques [57]. Finally, as shown in neuroeconomic studies in humans, providing instructions to subjects or making them learn more about the nature of decision variables may result in different choice biases [70]. A similar phenomenon might apply to social decisions. By contrast to protocols used with animal models, most testing of human prosociality involves paradigms in which information about social values is inevitably provided to the participants at the beginning of the experiment. One could hypothesize that human subjects might present reduced prosociality in protocols in which social values were experienced in a very novel situation and in which they could only be learned by trial and error.

## Data accessibility

Data are available online (https://doi.org/10.5281/zenodo.3911263).
